# Echolocation in the bat, *Rhinolophus capensis*: the influence of clutter, conspecifics and prey on call design and intensity

**DOI:** 10.1242/bio.201511908

**Published:** 2015-05-18

**Authors:** Kayleigh Fawcett, David S. Jacobs, Annemarie Surlykke, John M. Ratcliffe

**Affiliations:** 1 Sound and Behaviour Group, Department of Biology, University of Southern Denmark, Campusvej 55, DK5230 Odense M, Denmark; 2 Department of Biological Sciences, University of Cape Town, 7701 Rondebosch, South Africa; 3 Department of Ecology and Evolutionary Biology, University of Toronto, 25 Willcocks Street, ON M5S 3B2, Canada; 4 Department of Biology, University of Toronto Mississauga, 3359 Mississauga Road, Mississauga, ON L5L 1C6, Canada

**Keywords:** Bats, High-duty cycle echolocation, Jamming, Acoustic interference, Intensity

## Abstract

Echolocating bats are exposed not only to the echoes of their own calls, but often the signals of conspecifics and other bats. For species emitting short, frequency modulated signals e.g. vespertilionoids, adjustments in both the frequency and time domain have been observed in such situations. However, bats using long duration, constant frequency calls may confront special challenges, since these bats should be less able to avoid temporal and frequency overlap. Here we investigated echolocation call design in the highduty cycle bat, *Rhinolophus capensis*, as bats flew with either a conspecific or heterospecific in a large outdoor flight-room. We compared these recordings to those made of bats flying alone in the same flight-room, and in a smaller flight room, alone, and hunting tethered moths. We found no differences in duty cycle or peak frequency of the calls of *R. capensis* across conditions. However, in the presence of a conspecific or the vespertilionoid, *Miniopterus natalensis*, *R. capensis* produced longer frequency-modulated downward sweeps at the terminus of their calls with lower minimum frequencies than when flying alone. In the presence of the larger high-duty cycle bat, *R. clivosus*, *R. capensis* produced shorter calls than when flying alone or with a conspecific. These changes are similar to those of vespertilionoids when flying from open to more cluttered environments. They are not similar to those differences observed in vespertilionoids when flying with other bats. Also unlike vespertilinoids, *R. capensis* used calls 15 dB less intense in conspecific pairs than when alone.

## INTRODUCTION

Echolocating bats often fly in conspecific and heterospecific groups, especially at roosts and foraging sites Surlykke and Moss, 2000 Simmons, 2005. In such situations, bats receive not only the echoes of their own calls, but also calls and echoes originating from other bats. In such situations an individual bat has to contend with this barrage of relevant and irrelevant acoustic signals, and attend to only relevant signals while avoiding the potential problems of interference or jamming. How bats deal with such challenges has received most attention in species producing frequencymodulated (FM) echolocation signals at low duty cycles (<25) (see Ratcliffe et al., 2004; Ulanovsky et al., 2004 for review). Duty cycle is the percentage of time a bat is producing sound. Bats that use FM-calls and low duty cycles (LDC) have broadly tuned hearing ranges, reflecting the frequencies present in their calls (Moss and Schnitzler, 1995). Among them, molossids and vespertilionids (Vespertilionoidea) are the most studied with respect to echolocation behaviour in proximity to conspecifics (e.g., Miller and Degn, 1981; Obrist, 1995; Ratcliffe et al., 2004; Ulanovsky et al., 2004; Gillam et al., 2007; Chiu et al., 2010; Necknig and Zahn, 2011; Fawcett and Ratcliffe, 2015). However, phyllostomids and noctilionids have also recently been considered (Brinkløv et al., 2009; Dechmann et al., 2009).

Molossids and vepsertilionids emit biosonar signals through their mouths, producing calls that sweep downwards in frequency, often over an octave or more, are usually only a few milliseconds in duration, with more energy put into the lower frequencies of the sweep (Simmons et al., 1979; Schnitzler and Kalko, 2001). These and most other laryngeal echolocating species are believed to be unable to tolerate call-echo overlap as a result of reduced sensitivity in the middle ear synchronized to the emission of the call (Jen and Suga, 1976). Some of these LDC species have been reported to change the frequency of maximum energy (peak frequency, PF) of their echolocation calls when they encounter a conspecific using the same or similar PF (Obrist, 1995; Ratcliffe et al., 2004; Bartonička et al., 2007; Surlykke and Moss, 2000). In general, when a change in PF was noted, frequency was shifted upwards, not downwards (Gillam et al., 2007; Chiu et al., 2010; Bartonička et al., 2007). An upward shift may have an as yet unknown functional advantage, or it may reflect that in open space these bats are already using the lowest frequencies available to achieve maximum sonar detection range and minimize extraneous echoes (see Jakobsen et al., 2013). Shifting peak frequency away from that of the other bat reduces frequency and bandwidth overlap between individuals and may reduce interference (Obrist, 1995; Gillam et al., 2007; Bartonička et al., 2007).

Including those mentioned above, most echolocating bats (ca. 850 of ∼1100 spp.) are LDC echolocators (Fenton and Ratcliffe, 2010). The remaining laryngeal echolocating species use much longer calls of mostly constant frequency (CF), with short intercall intervals, resulting in high duty cycle (HDC) echolocation (reviewed in Schnitzler and Denzinger, 2011; Fenton et al., 2012). CF bats comprise the families Rhinolophidae and Hipposideridae, and a single mormoopid species (*Pteronotus parnellii*). CF bats use echolocation in much the same way as FM bats: both measure the time elapsed from call emission to echo return to judge the distance to an object, and time and intensity differences at the two ears to determine direction (Schnitzler and Denzinger, 2011; Fenton et al., 2012). In contrast to FM bats, however, CF bats discriminate between flying prey and background clutter using acoustic “glints” and Doppler shifts (Schnitzler and Denzinger, 2011; Fenton et al., 2012).

Glints (i.e. sudden increases in echo amplitude) correspond to the short period in the wing beat cycle where the surface of the wing is perpendicular to the bat's sound path. The Doppler shift of echo frequency results from the movement of the wing increasing and decreasing the frequency of the returning echo as they beat toward and away from the bat (Schnitzler and Denzinger, 2011; Fenton et al., 2012). Furthermore, the overall spectrum of the echo is shifted upward due to the bat's own flight speed, separating pulse and echo in the frequency domain. In contrast to FM bats, these shifts in frequency, rather than time, allow CF bats to tolerate call-echo overlap (Schuller, 1974, 1977). CF bats compensate for flight-induced Doppler shifts by adjusting call frequency such that the echoes fall into a narrow range of frequency sensitivity (Schnitzler, 1968; Simmons, 1974; Gaioni et al., 1990; Schnitzler and Denzinger, 2011).

This sensitivity is reflected in the bat's cochlea and onwards through the brain's auditory processing areas these components of the CF bat's auditory system are referred to as the auditory fovea and foveal areas, respectively (Rübsamen et al., 1988; Schnitzler and Denzinger, 2011). The combination of long CF calls, high duty cycle emission, the cochlear auditory fovea and associated foveal regions of the brain, enable CF bats to distinguish fluttering prey from non-fluttering objects (Schnitzler and Denzinger, 2011). This adaptive suite allows CF-bats to hunt for airborne prey in highly cluttered environments, a niche unavailable to most FM-bats (Schnitzler and Denzinger, 2011; Fenton et al., 2012). However, due to these adaptations, CF bats almost certainly do not have the same freedom to shift frequency when flying close to conspecifics as FM bats (Jones et al., 1994). Supporting this notion are results of studies of hipposiderids (Gustafson and Schnitzler, 1979; Jones et al., 1994) and rhinolophids (Jones and Rayner, 1989 these suggest it is unlikely that CF bats are able to shift frequency. We thus expect that CF bats do not have the option of shifting frequency available to them when flying with conspecifics because to shift frequency up (or down), even slightly beyond what is required for Doppler shift compensation, might render a CF bat insensitive to its own echoes.

When determining object range, CF bats are thought to measure the time elapsed between call and echo by using the frequency-modulated downward sweep that characterizes the end of their calls (Schnitzler, 1968; Schuller et al., 1971; Neuweiler et al., 1987; Schnitzler and Denzinger, 2011). Together with the much longer and extremely constant frequency in the middle and short upward FM sweep in the beginning, these three components give the CF bat echolocation call its well-documented staple-like shape. CF bats increase duty cycle when they encounter potential airborne prey, which should increase the bat's chances of detecting glints from insect wings and improve prey discrimination (Jacobs et al., 2008; Schnitzler and Denzinger, 2011; Fenton et al., 2012). Among CF bats, rhinolophids appear to be the most sophisticated echolocators; they have the most sharply tuned auditory fovea (Neuweiler, 1990; Schnitzler and Denzinger, 2011; Fenton et al., 2012) and use calls of longest duration and most constant frequency, at greater duty cycles than hipposiderids and *P. parnellii*.

**Fig. 1.**
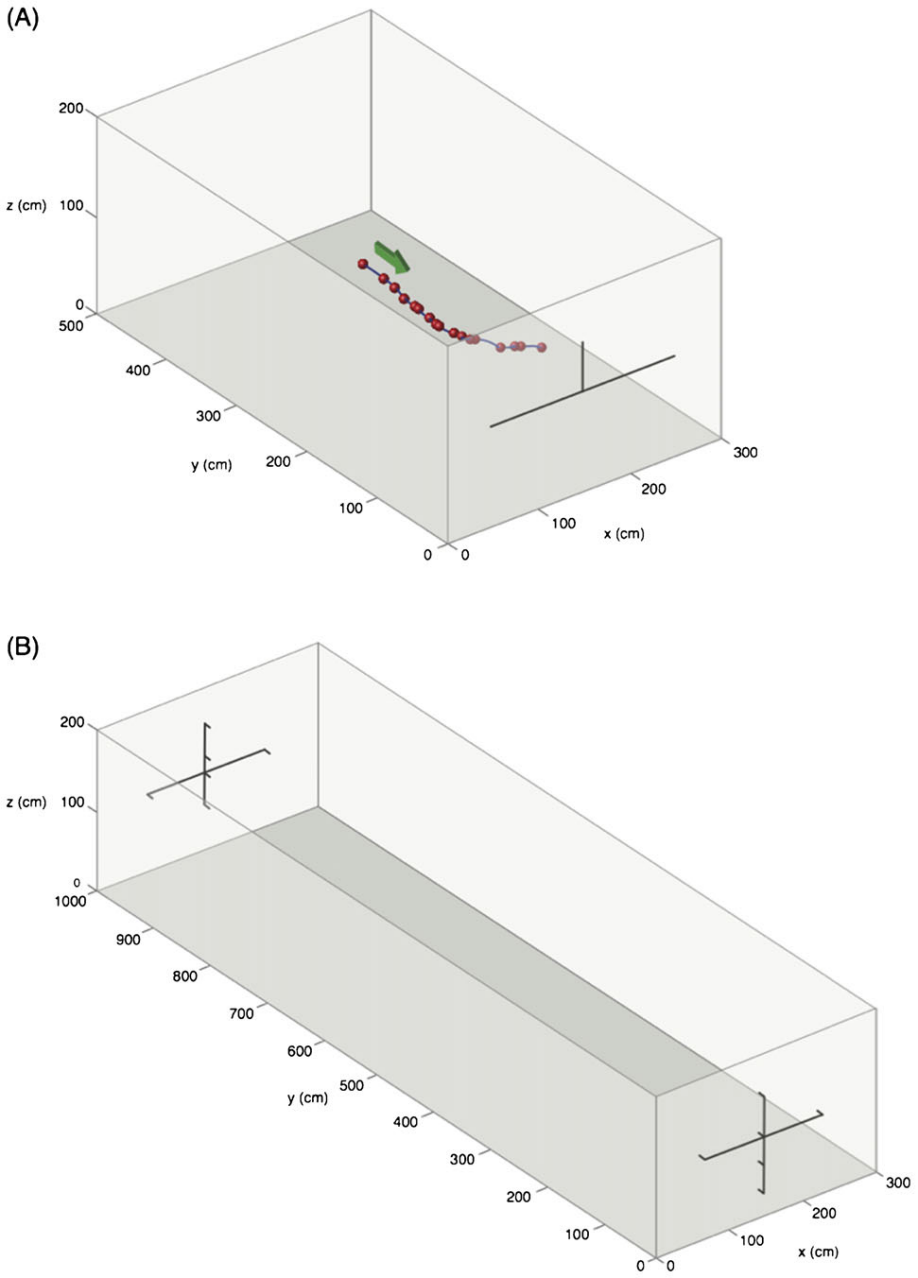
Flight rooms. (A) Small flight room (length 5 m × width 3 m × height 2 m) with flight path of a single *R. capensis* and (B) large flight room (length 10 m × width 3 m × height 2 m).

In this study we recorded the echolocation calls of the CF bat, *Rhinolophus capensis* (CF component=82–84 kHz at our study site) flying under a number of different acoustic conditions. In a large outdoor flight room (Fig. 1), we flew individual *R. capensis* alone, in conspecific pairs, and in heterospecific pairs with the CF bat, *Rhinolophus clivosus* (CF component=90–92 kHz and the FM bat, *Miniopterus natalensis* [PF=59 kHz; minimum frequency (–10 dB from PF)=52 kHz; maximum frequency (–10 dB from PF)=85 kHz]. In the wild, *R. capensis* is often found roosting with these two species (Schoeman and Jacobs, 2008). We also flew *R. capensis* alone in a small indoor flight room, with and without a tethered moth present. We did this to document the bats' echolocation behaviour (i) under more cluttered conditions and (ii) while tracking a moving target. We recorded the echolocation calls of the bats in all conditions using multi-microphone arrays to later measure call duration, call period, and frequency components, and to reconstruct flight paths and estimate call intensity. We predicted that, should *R. capensis* need to change its echolocation calls to operate in the presence of conspecifics, it would alter the characteristics of the FM components of their calls (i.e. increase duration and/or lower the minimum frequency) and/or increase call duration and duty cycle. We also predicted that the frequency of the CF component would not differ between conditions, reflecting the inflexibility of the highly tuned and individual specific auditory fovea of this species.

## RESULTS

### Echolocation behaviour of single bats in small room

**Fig. 2.**
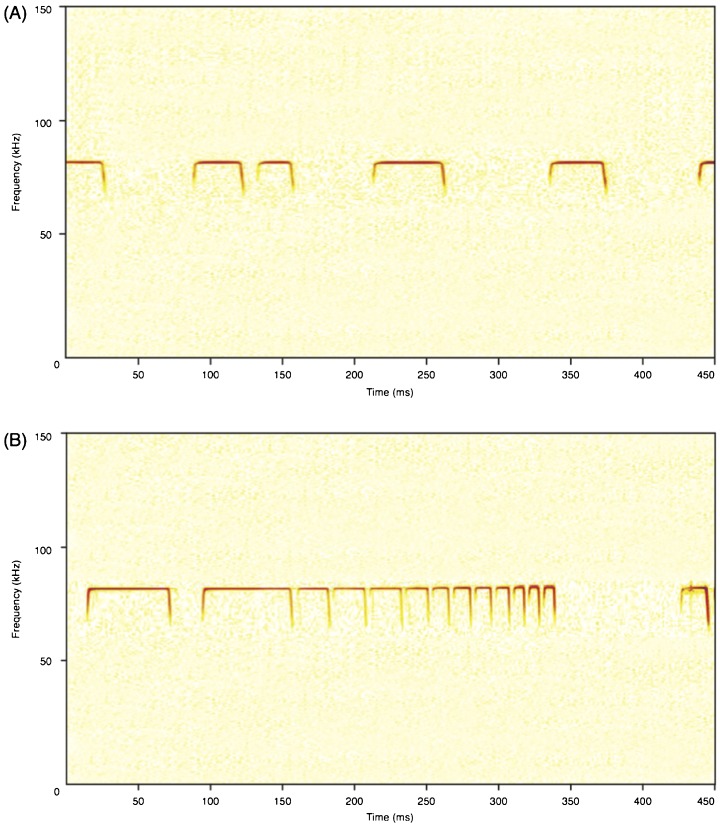
Echolocation call sequences of R. capensis during flight in this study. Calls emitted were of high duty cycle, and contained the characteristic long central CF call component, initiated by an upward FM sweep, FMi, and terminated by a downward FM sweep, FMt. (A) *R. capensis* flying alone in the large flight room. (B) *R. capensis* capture sequence as it attacks a moth when flying alone in the small flight room.

When flying alone in the small flight room, *R. capensis* (N=5 bats) emitted calls (Fig. 2A) with an average PF of 84.2 kHz. Average call duration was 33.6 ms (minimum=22.8, maximum=58.1); average period was 73.3 ms (n=5, minimum=52.9, maximum=99.5), resulting in an average duty cycle of 45.8%.

When presented with a tethered moth in the same small flight room, *R. capensis* (*N*=5) always initiated an attack and emitted a distinctive echolocation sequence. In this situation, bats produced ca. 50 ms calls early in the call sequence (see Fig. 2B), gradually shifting to shorter call durations and periods. Duty cycle increased over the course of an attack right up until the buzz, from 66% in the beginning of a capture sequence up to 84% immediately before the buzz (*N*=5). The buzz (i.e. call rate > 100 calls/s) had a duty cycle of 70–90% (based on two bats). Over the course of attack, the bat lowered the lowest frequency of the terminal sweep (Fig. 2B) of the FMt component. Using intensity measures from the centre microphone (ca. 50 cm behind the moth), as expected, we found that the bats reduced the intensity of their echolocations signals as they closed in on their prey. Pre-buzz calls were had a median intensity of 101.5 dB [range: 71–112]; buzz calls had a range of 80–95.4 dB [median: 87].

### Echolocation behaviour in large room

Call peak frequency, period, and duty cycle did not differ significantly between groups (Table 1, for group descriptions, please refer to Methods). FMi duration and minimum (–10 dB from PF) frequency were also not significantly different (Table 1). Similarly, individual range (max-min) in call duration and call period was not significantly different across conditions (P–0.7). Significant differences were found between groups for other parameters, and these differences are described in Table 1.

**Table 1.**
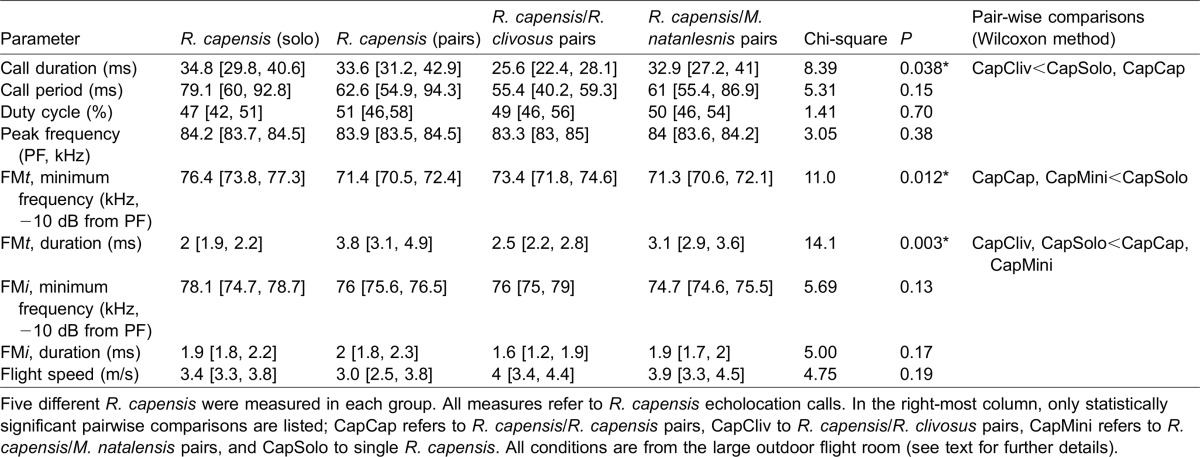
Echolocation call characteristics and flight speed (group mean [group range])

#### Single bats – large flight room

When flying alone in the large outdoor flight cage, *R. capensis* emitted characteristic long staple-shaped calls with a short upwards FM sweep at the start of the call (FMi) followed by a long CF component ending with a short downward FM sweep (FMt) (for details see Table 1). Maximum call intensity was 123.7 dB SPL (n=5, minimum=107.9 dB SPL, range=15.8).

#### Conspecific pairs

Minimum frequency in FMt was significantly lower in *R. capensis* when flying with a conspecific than when flying alone or with *R. clivosus* (Table 1). Correspondingly, FMt duration was significantly longer in *R. capensis* when flying with a conspecific than when flying alone or with *R. clivosus* (Table 1).

Sufficient intensity data for statistical comparison were only obtained for solo *R. capensis* flights and *R. capensis*/*R. capensis* pairs which we compared using the Wilcoxon rank-sum test.

When flying alone, *R. capensis* produced more intense echolocation signals than when flying with a conspecific [two sample t-test, n=10, P<0.05; *R. capensis* (solo): mean=115 dB SPL, range=108–124; *R. capensis* (pairs) mean=100 dB SPL, range=91–106)].

#### Heterospecific pairs – *R. clivosus*

Call duration of *R. capensis* flying with *R. clivosus* was significantly shorter than for *R. capensis* flying solo, in conspecific pairs or when paired with *M. natalensis* (Table 1), for comparison sake, there was no difference between *R. capensis* flying alone and in conspecific pairs.

#### Heterospecific pairs – *M. natalensis*

Minimum frequency in FMt was significantly lower in *R. capensis* when flying with *M. natalens*is than when flying alone or when flying with *R. clivosus* (Table 1). FMt duration was significantly longer in *R. capensis* when flying with *M. natalensis* than when flying alone or flying with *R. clivosus* (Table 1). We found no other significant differences between groups.

## DISCUSSION

**Fig. 3.**
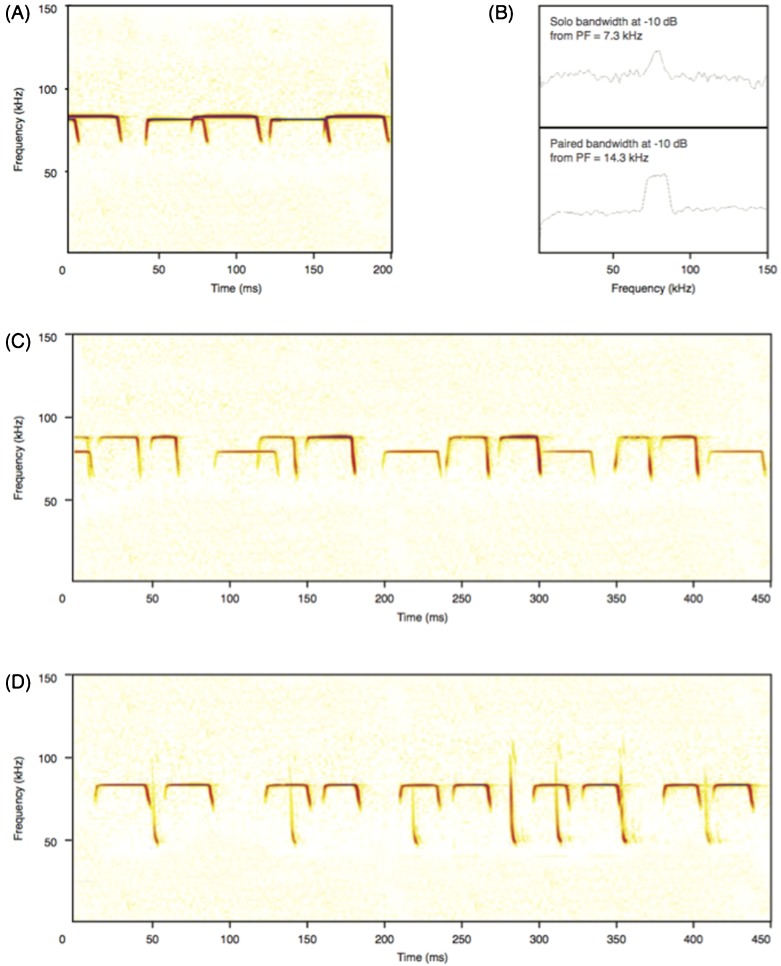
Echolocation calls. (A) R. capensis flying in a conspecific pair in the large flight room. (B) Power spectra (fast Fourier transforms) of FMt for *R. capensis* flying alone (top panel) and flying in a conspecific pair (bottom panel), both in the large flight room. (C) *R. capensis* flying with *R. clivosus* in the large flight room. (D) *R. capensis* flying with *M. natalensis* in the large flight room.

In this study, we used multi-microphone arrays to investigate the echolocation behaviour of *R. capensis* under different acoustic conditions. Specifically, we examined the potential perceptual challenges this species might face when echolocating in the presence of conspecifics and heterospecifics. To assess this, we examined the differences in the echolocation calls of *R. capensis* under six different conditions. As predicted, we found no frequency differences in the CF component of *R. capensis* echolocation calls, but we did find differences in the temporal and spectral structure of the FMt when this bat flew with conspecifics and with a heterospecific, *M. natalensis* as compared to *R. capensis* flying alone in the same large flight room (Fig. 3). In this same room, we also found that *R. capensis* produced calls of shorter duration when it flew with *R. clivosus* as compared to when flying alone (Fig. 3).

As Jones et al. (Jones et al., 1994) previously reported for hipposiderids, we found no significant differences in the frequency of the CF component across groups and conditions. As CF bats approach an object, for a given emitted frequency, the returning echo will be of higher frequency (due to Doppler-shift). CF bats, and especially rhinolophids, are able to compensate for these changes in frequency by emitting lower frequency calls resulting in returning echoes of a specific frequency (Schnitzler, 1967; Schnitzler, 1968; Schnitzler, 1973; Konstantinov et al., 1978; Tian and Schnitzler, 1997; Hiryu et al., 2008; Schnitzler and Denzinger, 2011). This frequency specificity is reflected in the cochlea and auditory regions of the brain and ensures the bat is very sensitive to echoes returning at the frequency maintained through Doppler-shift compensation, but is quite insensitive to frequencies just outside this very narrow tuning (Neuweiler, 1970; Schnitzler et al., 1971; Schnitzler, 1976; Schnitzler and Denzinger, 2011). Auditory insensitivity outside this specific and narrow frequency range renders the bat almost deaf to its own calls, and may mean that changing its emitted CF frequency in conspecific situations, for example to avoid jamming, might make the bat insensitive to its own echoes.

However, due to this high specificity in frequency sensitivity, *R. capensis* may be as insensitive to the calls of conspecifics as it is to its own. Furthermore, individual rhinolophids have ‘ personal’ resting frequencies for the CF component of their echolocation signals and ‘ personal’ reference frequencies (roughly 200 Hz higher than resting) in the auditory fovea and foveal areas of the brain (Schnitzler and Denzinger, 2011; Furusawa et al., 2012). Here we found an average emitted frequency of 84.2 kHz ranging from 83.7 to 84.5 kHz. Since we selected calls from bats approaching the array with average flight speeds of 3.4 m/s we have over-estimated the emitted frequency by approximately 0.8 kHz due to the Doppler-shift towards the microphone. The returning echo is also Doppler shifted by approximately another 0.8 kHz, so the bats here receive echoes from objects straight ahead with frequencies ranging from approximately 84.5 to 85.3 kHz. Furthermore, the calls of conspecifics are probably not a problem if the bats fly away from one another due to the directionality of the sound beam (Matsuta et al., 2013) and, more than this, will be negatively Doppler shifted. When two bats fly in parallel (tandem flight) there will be no Doppler shift.

As a result, in these two situations rhinolophids may be quite insensitive to the calls of conspecifics. However, if bats approach one another, the conspecific call will be doubly Doppler shifted (like the echo) due to the flight velocity of both bats, and it is likely the conspecific call will fall right in the most sensitive part of the acoustic fovea. Unfortunately, we did not have enough flight paths to separately test these three flight situations (flying away, parallel and against). However, the strict constraints from the tuning of the acoustic fovea is likely to force CF bats to emit call frequencies adjusted to the relative velocities of the objects it approaches, while leaving little to no freedom for frequency changes, which is corroborated by our results showing no significant difference in emitted CF across any of the conditions. Hence, our results support our hypothesis that even if conspecific calls jam rhinolophid echolocation, these bats cannot compensate by using a jamming avoidance response in the frequency domain, which would involve further, potentially maladaptive adjustments to the emitted CF-frequency.

We did, however, find other differences in the temporal and spectral structure of the calls of *R. capensis* flying with other bats (Table 1). *R. capensis* emitted shorter calls in the presence of *R. clivosus* compared to *R. capensis* flying alone or with conspecifics, but maintained roughly constant duty cycle (50%) across all situations (Table 1). This shorter duration in the presence of a larger heterospecific with whom *R. capensis* overlaps in diet (Jacobs et al. 2007) suggests a number of plausible explanations, although none are strongly supported by our data and none excludes another. First, *R. clivosus* is larger than *R. capensis* and may represent a potential physical threat. There is evidence that rhinolophids, including *R. capensis*, recognize other rhinolophid species from the CF component of their echolocation calls (Schuchmann and Siemers, 2010). If so, once *R. clivosus* is recognised by these echolocation cues, *R. capensis* may switch to using shorter call durations to increase the production of FMt sweeps, which may allow *R. capensis* to better estimate its distance from and possibly track *R. clivosus*. If so, this reaction may have social significance for these two rhinolophids, which roost together at our study site.

Alternatively, or additionally, *R. capensis* may be reacting to *R. clivosus* simply as “flying clutter” or a moving obstacle to negotiate in the same airspace. Shorter call durations and call periods lead to an increase in the number of FMt produced per unit time. FMt may play a key role in distance estimation to both stationary and moving objects, as we discuss later. If so, taken together, these changes could suggest that *R. clivosus* may be interpreted by *R. capensis* as a moving object whose position in space is being updated frequently. However, why these differences are observed when flying with *R. clivosus* yet not when flying with conspecifics or flying with the vespertilionid, *M. natalensis* is unclear.

Although *R. capensis* in the presence of conspecifics and *M. natalensis* showed no difference in call duration compared to *R. capensis* flying alone, they did produce calls with longer FMt sweeps, with lower minimum frequencies than when flying alone or with *R. clivosus*.

These changes also suggest that *R. capensis* may interpret these other bats as clutter or moving objects. The FMt sweep may be used by CF-bats as it is by FM-bats i.e. to precisely estimate the distance to objects, moving and stationary (Schnitzler, 1968; Schuller et al., 1971; Schnitzler and Denzinger, 2011). However, Novick (Novick, 1971) argued that rhinolophids might be able to estimate object distance based on call-echo overlap using Doppler-shift induced frequency changes in the CF component, but provided little justification for his argument. Others suggest that the FMt is a by-product of production of the CF call component (Schnitzler, 1968; Tian and Schnitzler, 1997). Our data contradict these latter arguments and support the former. We show that *R. capensis* decreases FMt minimum frequency when in proximity to a moving target, whether it be a moth or bat of similar size. This corroborates the functional significance of FMt of the rhinolophid echolocation call. The role of FMt in obstacle avoidance and inflight tracking is further supported by observations of perched rhinolophids not including FM-components (Schnitzler et al., 1985; Neuweiler et al., 1987).

We found that call intensity was higher in *R. capensis* flying alone, when compared to when flying in conspecific pairs. This reduction in intensity may also reflect a clutter or obstacle- like reaction to other bats in proximity. Alternatively it could represent an anti-interference reaction. In more cluttered habitat, some FM-bats use lower on-axis intensity; some broaden their sonar beams (Surlykke et al., 2009; Jakobsen et al., 2013), while others narrow their beam, perhaps as a means of clutter rejection (Brinkløv et al., 2011). Regardless, by lowering on-axis intensity the calls of a given bat at a given distance would also be less detectable to conspecifics in proximity than had the bat maintained on-axis intensity at solo levels. Whether it is a “noble” behaviour of the bat to not disturb its conspecifics or it is simply a reaction to objects close by the effect is the same: reduction of interference from the other bats' calls. Brinkløv et al. (Brinkløv et al., 2009) found lower call intensity in *Macrophyllum macrophyllum* flying in groups (95 dB SPL at 10 cm from the bat's mouth) compared to when flying alone (101 dB) in a flight room, and in both cases substantially lower than when flying in the open in the field (111 dB). The function or functions of lowered call intensity in CF-bats in the presence of conspecifics also suggests that bats may experience one another as additional clutter. We note that the observed reaction is the opposite of the Lombard response (i.e. increasing intensity in response to increased background noise), which has been observed in the FM bat, *Tadarida brasiliensis* (Tressler and Smotherman, 2009).

Rhinolophids and hipposiderids emit echolocation calls through their two nostrils (Schnitzler and Grinnell, 1977). The observed 15 dB lower call intensity in conspecific pairs compared to *R. capensis* flying alone indicate that in the presence of conspecifics, this species reduces the maximum range of its echolocation, or the space it ensonifies in the forward direction. In mouth-emitting vespertilionids, a reduction in on-axis intensity is accompanied by a broadening of the beam, both of which have been attributed to operating in clutter and a means of obstacle detection (Jakobsen et al., 2013). It is hard to imagine how a nostril emitting CF bat would broaden the beam, since emitter size and frequency are presumably fixed, however a recent study on another species of rhinolophid bat *R. ferrumequinum* shows that sonar beam breadth, while narrower than that of vespertilionids (Surlykke et al., 2009; Jakobsen et al., 2013), is broadened at least somewhat during the final phase of an aerial attack (Matsuta et al., 2013).

Taken together, these differences in echolocation call behaviour of *R. capensis* in the presence of other bats compared to when flying alone, are not easily reconciled with previous reports from FM bats and deserve further attention under more controlled conditions. We speculate that the bats may be actively tracking the physical bodies of bats in proximity, a supposition supported by our visual observations. However, the fact that *R. capensis* reacted differently to *R. clivosus* compared to conspecifics and *M. natalensis* may indicate that it is not just the physical body of the other bat, but also its echolocation calls that determines the changes observed in *R. capensis* echolocation calls. We found no difference between how *R. capensis* reacted to a conspecific versus the distantly related, but similarly sized, FM-bat *M. natalensis*. In both of these situations, *R. capensis* produced calls with longer FMt duration and lower minimum FMt frequency, compared to when it flew alone. These are similar to the call designs observed when *R. capensis* is attacking prey, and flying in the smaller room (Fig. 2). These FMt changes are also reminiscent of the modifications FM-bats make to their calls in the face of increasing habitat complexity (Obrist, 1995; Schnitzler and Kalko, 2001). For FM bats, calls with broader call bandwidths may be able to obtain more details about the distance, size, shape and velocity of the target it is attending to by virtue of having more listening frequencies available to it (Simmons and Stein, 1980) and this may also be the case with the FM components of rhinolophids calls. The shorter calls used by *R. capensis* in the presence of *R. clivosus* is also suggestive of a clutter reaction see (Schnitzler and Kalko, 2001).

In conclusion, the differences we found in FMt bandwidth, call duration, and call intensity point to a reaction to other bats as simply obstacles. Our results also support the function of the terminal FM-sweep for accurate ranging. Based on assumptions drawn for FM bats, none of the differences observed in the presence of another bat compared to when flying alone is unambiguously indicative of an attempt to minimize jamming or interference, suggesting that rhinolophids may pay for their sophisticated Doppler compensation capability by an apparent lack of flexibility in the constant frequency component of their calls. However, this physiological constraints argument ignores the possibility that CF bats, and plausibly all echolocating bats, are able to filter their signals from those of other bats using auditory neurons at the periphery and, if necessary, high-order auditory processing using mechanisms as yet undiscovered in the bat's brain. Similarly, it may be that differences in CF versus FM bat echolocation mean that what increases interference in FM bats (e.g. greater bandwidth) may improve the situation for CF bats when flying in conspecific groups.

## MATERIALS AND METHODS

### Study site

This study was conducted at De Hoop Nature Reserve, near Bredasdorp, Western Cape, South Africa, an area characterized by fynbos vegetation (i.e. low growing, sclerophyllous vegetation).

We captured bats at two cave sites at De Hoop: Guano Cave and Hothole Cave. Guano Cave is a roosting site for *Rhinolophus clivosus*, *Myotis tricolor* and *Miniopterus natalensis*. Hothole cave is a roosting site for *R. capensis*, *R. clivosus*, *My. tricolor* and *Mi. natalensis*.

### Bats

We caught a total of 29 adult bats for this study: 21 *R. capensis* (10 male, 11 female), 5 *R. clivosus* (all female), 2 *Mi. natalensis* (both male). We caught *R. clivosus* using hand nets at Guano Cave and *R. capensis* and *M. natalensis* using mist nets near to Hothole Cave. After each recording session we released the bats at site of capture either immediately after trials or within their roost the following day. All bats were released within 24 hours of capture.

This research was approved by the Science Faculty Animal Ethics Committee of the University of Cape Town (2011/v6/DJ) and conducted under permit number 0035-AAA004-00626 from Cape Nature, Western Cape Province, South Africa.

### Flight rooms

We flew bats in one of two flight rooms see (Fig. 1). One was an outdoor netted cage (length 10 m × width 3 m × height 2 m) previously described in Jacobs et al. (Jacobs et al., 2008); the other was smaller and had concrete walls, floor and ceiling (length 5 m × width 3 m × height 2 m), which we lined on the inside with a large net matching the room dimensions. In the large room, we allowed single bats, conspecific pairs (*R. capensis*/*R.capensis*), and heterospecific pairs (*R. capensis*/*R. clivosus*; *R. capensis*/*Mi. natalensis*) to fly freely. In all, there were four large room conditions (solo and three species-pair combinations). Bats tested under one large room condition were not tested under any other.

In the smaller room, we flew individual *R. capensis* alone and in the presence of a tethered moth. Apart from the hunting trials in the small room, bats were not fed during trials.

### Recordings

We used multi-microphone arrays to record bats in flight. In pair situations we recorded sound files only when both bats were flying.

In the large room, we used cross-shaped arrays, either one with 12 microphones at one end of the room or two with 6 microphones positioned at each end of the flight room see (Fig. 1).

In the smaller room, we used an inverted T-shaped array of 4 microphones. This array was placed at one end of the room, with the centre microphone 1 m from the ground, level with and on-axis to the moth, which was tethered 1 m in front of it.

All arrays were covered in sound-absorbing cotton batting, with the microphones protruding roughly 10 cm from the array frame, well past the cotton batting.

Each multi-microphone array used a combination of two kinds of microphones, 1/4″ 40 BF G.R.A.S. microphones (grids off) (G.R.A.S. Sound and Vibration Measurement A/S, Holte, Denmark) and Avisoft CM16 condenser microphones (Avisoft, Berlin, Germany). In the small room, the G.R.A.S. microphones were positioned in a horizontal line, 60 cm apart with an Avisoft microphone 30 cm directly above the middle G.R.A.S. microphone (Fig. 1). In the large flight room, two cross-shaped multi-microphone arrays, one positioned at each end of the flight room, were also composed of both 1/4″ 40 BF G.R.A.S. microphones (grids off) (G.R.A.S. Sound and Vibration Measurement A/S, Holte, Denmark) and Avisoft CM16 condenser microphones (Avisoft, Berlin, Germany). Sounds recorded using Avisoft microphones were not used for intensity calculations, only for 3D positioning. Intensity estimates are given as dB RMS at 10 cm from the bat's moth (Surlykke et al., 2009).

G.R.A.S. microphones were calibrated to a 1 kHz pure tone at 94 dB SPL with a Bruel and Kjr calibrator (type 4231, Bruel and Kjær, Naerum, Denmark) and were amplified using G.R.A.S. amplifiers (30 dB amplification, high pass filtered to 15 kHz). Signals were sampled with an Avisoft USGH 1216 A/D converter at a sample rate of 300 kHz per channel (16-bit). Our files were stored onto a laptop using Avisoft Recorder USGH software. In the large room, we recorded files of 3 seconds in duration (2 second pre-trigger, 1 second post-trigger time). In the small room, we used 2 seconds pre-trigger, 2-seconds post-trigger.

### Flight path reconstruction

We used a custom Matlab script (by L. Jakobsen, Lund University) to obtain the 3D coordinates of the bats as they emitted each echolocation call. These were calculated by determining the time-of-arrival differences at the different microphones by cross-correlating the signals across multiple channels. We determined the spatial position of bats by filtering out the CF component of their calls, and using only the frequency-modulated components. We then used these 3D coordinates to create a visual representation using a second custom Matlab script (by B. Charlton, University of Southampton), which allowed us to visually screen files and select only those with unambiguous flight paths for sound analysis. In pair situations, files were only selected for further analysis when flight paths showed that both bats were flying simultaneously.

### Sound analysis

From the selected recordings with unambiguous flight paths, we split the multi-channel wav files into single channel wav files and screened them using Adobe Audition (Adobe Systems Inc., San Jose, CA). From the files selected during this screening process, we then analysed call sequences with good signal to noise ratio (signal amplitude > 10 dB than noise) in BatSound (v. 4, Pettersson Elektronik AB, Uppsala, Sweden). Frequencies of the CF part were analysed with 1024-point FFT window. The FM parts in the beginning and the end of each call, FMi and FMt, respectively, were analysed with a 256-point FFT window. Time parameters (call duration, call period) were measured from oscillograms. Call period refers to the time elapsed from the start time of the focal call to the start time of the next call. Duty cycle was calculated by dividing average call duration by average call period for each bat.

From the power spectrum [fast Fourier transform function (FFT) size 1024, Hann window] of each call we measured peak frequency (PF). We also measured the minimum frequency (–10 dB from PF) of the FMi and FMt sweeps.

### Statistical analyses

We used JMP v. 10.0 (SAS Institute, Cary, NC, USA) for all statistical analyses. All tests were two-tailed (+0.05). Our data did not meet all of the assumptions of ANOVA, therefore we used equivalent non-parametric tests. We compared call peak frequency, duration, period, duty cycle, and the duration and minimum frequency of FMi and FMt across the four trial types in the large flight room (*R. capensis* solo, *R. capensis*/*R. capensis* pairs, *R. capensis*/*R. clivosus* pairs, *R. capensis*/*M. natalensis* pairs) using Kruskal-Wallis tests (rank-sums) and, if significant, non-parametric comparisons for each pair using the Wilcoxon method.
